# Financial Knowledge or Managerial Competence? Disentangling Financial Literacy and Liquidity Constraints for Processing Continuity and Food Security in the Turkish Tea Industry

**DOI:** 10.3390/foods15122139

**Published:** 2026-06-13

**Authors:** Musa Gün, Mustafa Savcı

**Affiliations:** Faculty of Economics and Administrative Sciences, Recep Tayyip Erdoğan University, Rize 53100, Türkiye; mustafa.savci@erdogan.edu.tr

**Keywords:** financial management competency, business liquidity, operational continuity, agro-food processing, tea processing sector, financial literacy, cash-flow constraints, processing resilience, Türkiye

## Abstract

The economic resilience of agricultural enterprises is increasingly relevant for maintaining processing continuity and food quality in highly perishable agro-food chains. This study examines the associations between financial knowledge, financial management competency, business liquidity, and operational food-processing continuity in Türkiye’s tea sector. A quantitative cross-sectional design was employed, using structured survey data from 203 senior managers across 86 public and private tea-processing firms in Rize Province. The data were analysed using Ordinary Least Squares regression, mediation analysis, exploratory factor analysis, and robustness checks in accordance with OECD/INFE guidelines. Results indicate a significant deficit in theoretical financial knowledge (mean score: 4.47/10) alongside widespread overconfidence among 85% of managers. Applied financial management competency is positively associated with perceived business liquidity (*β* = 0.336, *p* < 0.001), suggesting that practical budgeting, cash-flow planning, and financial decision-making capabilities are relevant to maintaining operational funding capacity. In contrast, cash-flow difficulties are not significantly explained by firm-level financial knowledge, managerial competency, liquidity, or ownership structure (R^2^ = 0.014, *p* = 0.722), indicating that these difficulties may reflect broader seasonal and sector-wide financing constraints. The findings challenge the assumption of a linear relationship between theoretical financial knowledge and managerial outcomes. They suggest a dual policy approach that combines applied financial management training with structural financing mechanisms to ensure the continuity of fresh leaf procurement and processing. While the study does not directly measure food safety, post-harvest losses, or SDG outcomes, the results have potential implications for reducing processing disruptions and supporting more resilient agro-food processing systems.

## 1. Introduction

The sustainability of agro-food systems is increasingly linked to the economic resilience of agricultural enterprises, which must maintain processing efficiency and food quality amid macroeconomic volatility, climate-induced disruptions, and structural financing constraints [1,2]. Within this framework, the tea sector holds considerable strategic importance—not only serving as a primary livelihood for millions of smallholder producers but also acting as a critical pillar of rural development in producing nations [3,4].

Türkiye is a vital actor in the global tea ecosystem, accounting for approximately 4–7% of global tea production and consistently ranking among the top five global producers [5,6,7]. Nationally, the sector is highly geographically concentrated, with Rize Province alone responsible for 65–68% of the country’s green tea output [8]. This concentration renders tea a strategic agricultural product that anchors regional socioeconomic stability and deters rural-to-urban migration—making it indispensable to Türkiye’s food security architecture [3,9].

Despite its strategic value, the tea processing sector is inherently burdened by long production cycles and seasonal revenue streams, which may increase the risk to food quality management [10]. Fresh tea leaves are highly perishable and require timely processing to prevent uncontrolled oxidation, preserve antioxidant properties (such as catechins), and ensure compliance with food quality and safety standards. However, tea processing enterprises operate with exceptionally low asset turnover rates and extended inventory holding periods, averaging up to 107 days, which severely lengthen the cash conversion cycle [6]. This structural cash-flow blockage may force enterprises into dependency on high-cost, short-term debt. Consequently, financial bottlenecks may delay leaf procurement and restrict investments in processing technologies, thereby increasing the risk of processing interruptions and quality deterioration in highly perishable tea-processing chains [11,12].

The operational resilience of tea-processing facilities is relevant to broader discussions on sustainable food systems, particularly SDG 2 and SDG 12, although the present study treats these goals as policy-relevant contextual frameworks rather than directly measured empirical outcomes. SDG 2 emphasizes food security, nutrition, and resilient agricultural systems, while SDG 12.3 targets reducing food losses along production and supply chains, including post-harvest losses. Food system resilience refers to the capacity of food systems and their units to continue providing sufficient, appropriate, and accessible food despite shocks and disturbances [13]. Similarly, local food system resilience is increasingly viewed as a key condition for protecting food security under economic, climatic, and institutional shocks [14]. In highly perishable agro-food chains such as tea, delays in the procurement and processing of fresh leaves due to liquidity constraints may pose an operational risk. Such delays may accelerate deterioration in quality and reduce the retention of bioactive compounds, such as catechins and polyphenols. Accordingly, strengthening financial resilience in tea-processing enterprises can be viewed as a potential mechanism for supporting responsible production and resilient agricultural value chains, rather than as direct evidence of improved food security or food loss outcomes [15,16,17,18].

Addressing these vulnerabilities requires understanding how financial capabilities are associated with processing efficiency within the food supply chain. Through the lens of the Knowledge-Based View (KBV) and Resource-Based View (RBV), financial literacy can be conceptualized as a strategic capacity that may help an enterprise plan liquidity, evaluate investments, and allocate resources for food-processing activities [19,20]. Nevertheless, the existing literature presents a critical gap: financial literacy is predominantly analysed at the general SME level, with limited attention to how financial constraints may translate into operational risks, such as delayed processing and compromised quality, in highly seasonal agricultural sectors [21,22,23]. Furthermore, prior studies have largely failed to conceptually separate theoretical financial knowledge from applied management competencies when evaluating an enterprise’s capacity to sustain operational continuity [24,25].

This study addresses these gaps by empirically examining the financial knowledge and financial management competencies of tea industry managers in Türkiye and by assessing their associations with business liquidity and payment-related operational food-processing continuity. By linking financial capability to liquidity and processing continuity, the study positions tea-sector financial management as a potential micro-level mechanism relevant to SDG-oriented agro-food policy. Given the quantitative and hypothesis-testing design of the study, the empirical analysis is guided by the following hypotheses:

**H1.**  *Financial knowledge and financial management competency differ significantly between public and private tea-processing enterprises.*

**H2.**  *Applied financial management competency is positively associated with perceived business liquidity.*

**H3.**  *Theoretical financial knowledge is not necessarily translated into applied financial management competency.*

**H4.**  *Financial management competency mediates the relationship between theoretical financial knowledge and business liquidity.*

**H5.**  *Firm-level financial knowledge, financial management competency, and liquidity are associated with operational food-processing continuity, while cash-flow difficulties may also reflect sector-wide structural constraints.*

The remainder of this study is structured as follows: Section 2 reviews the literature on sustainable food processing, financial literacy, food system resilience, and macroeconomic constraints. Section 3 details the empirical methodology. Section 4 presents the descriptive statistics, regression findings, and mediation analysis. Section 5 discusses the implications for food quality, processing continuity, post-harvest loss prevention, and SDG 2 and SDG 12. Section 6 concludes with policy recommendations to strengthen financial resilience, food security, and responsible agro-food production.

## 2. Literature Review

### 2.1. Macroeconomic Vulnerabilities, Processing Efficiency, and Food System Sustainability

Sustainable agriculture integrates ecological preservation, food quality maintenance, and economic viability into a holistic production framework [1]. Economic sustainability—defined by an enterprise’s capacity to maintain stable cash flows—is a prerequisite for broader food security goals, as it may influence a firm’s ability to invest in hygienic processing conditions and modern technology [2,25]. The tea sector is highly sensitive to financial risks, including inflation, interest rate volatility, and input cost spikes [12,26,27]. Structurally, agricultural enterprises suffer from low asset turnover and prolonged cash conversion cycles, which restrict operational cash flow and force firms to compromise on processing technologies, potentially increasing the risk of post-harvest deterioration or processing-related losses [6,28].

Managing these financial risks may support food safety-related operational continuity and uninterrupted processing [2,29]. Ref. [16] confirm that agricultural financing research is increasingly centered on food sustainability imperatives, while Ref. [30] demonstrate that integrated financial and operational management significantly enhances agricultural sector resilience and product quality. State-level financial support remains indispensable to prevent quality degradation caused by systemic constraints, such as delayed raw material procurement [31,32].

### 2.2. Conceptualization of Financial Literacy for Food Sector Operations

The academic discourse distinguishes between conceptual and operational definitions of financial literacy [33,34,35]. Conceptually, financial literacy represents the cognitive ability to understand financial mechanisms; operationally, it is measured through behaviors such as budgeting and investing in operational upgrades [33,34]. In agricultural contexts, measurement typically relies on numeracy, compound interest, inflation, and risk diversification [5,36,37].

Theoretical frameworks, including the KBV and RBV, elevate financial literacy to an intangible corporate resource that may help firms allocate resources for maintaining food quality standards despite market volatility [20,38]. However, empirical evidence indicates significant deficiencies; comprehension of inflation and risk diversification remains critically low among agricultural operators [9,39]. Ref. [15] explicitly link financial literacy to sustainable food production outcomes, demonstrating that literacy improvements may be associated with better resource allocation, enhanced processing capabilities, and reduced vulnerability to food insecurity.

### 2.3. Financial Literacy, Overconfidence, and Food Supply Chain Stability

High financial literacy may support budget management, enabling food processors to allocate funds to facility maintenance and to make timely raw material purchases, thereby reducing the risk of operational interruptions [40,41,42,43,44]. However, behavioral finance literature introduces the overconfidence bias [45,46]. Managers may exhibit high subjective confidence despite low objective knowledge, leading to suboptimal borrowing and underinvestment in essential processing infrastructure [45,47]. This ‘implementation gap’ suggests that knowledge alone does not guarantee sound operational management [48,49]. In food systems, managerial miscalibration may increase the risk of delayed supply-chain operations and inefficient resource allocation, particularly where processing continuity depends on timely procurement and working-capital planning.

### 2.4. Structural Constraints, FinTech, and Agricultural Food Sustainability

Stringent collateral requirements frequently impede access to credit to upgrade food processing facilities [7,39,50]. Digital financial inclusion—facilitated by FinTech and blockchain—offers mechanisms to bypass traditional barriers, enabling faster payments to farmers and preventing the degradation of raw materials waiting at procurement stations [51,52,53].

Ref. [3] show that adopting blockchain-based supply chain frameworks in the tea sector is associated with improvements in traceability and food safety. However, adopting these technologies requires sufficient Digital Financial Literacy to prevent unrecognized cyber risks and debt traps [47,54,55,56,57,58,59].

### 2.5. Financial Constraints, Post-Harvest Loss, and Processing Continuity in Agro-Industries

Financial constraints in agro-processing industries are not limited to profitability or firm survival; they may also affect a firm’s ability to maintain regular processing flows. Food system resilience is commonly defined as the capacity of food systems and their units to continue providing sufficient, appropriate, and accessible food despite disturbances [13]. In perishable agricultural value chains, insufficient working capital, delayed procurement payments, and interruptions in labor, energy, or maintenance financing may slow down processing activities [14]. This is particularly relevant for tea processing, where fresh leaves must be transformed shortly after harvest to preserve quality attributes [17]. However, the link between financial constraints and food-system outcomes should be interpreted with caution [18]. Most firm-level financial literacy studies do not directly measure physical food loss, biochemical quality degradation, or food-safety outcomes. Therefore, financial management competency should be understood as an indirect operational capability that may support processing continuity, rather than as direct evidence of reduced post-harvest losses or improved food security. This distinction motivates the present study’s focus on liquidity and operational food-processing continuity as intermediate firm-level outcomes [15,16].

### 2.6. Nutritional Preservation and Bioactive Compound Retention in Tea Processing

Tea processing is highly time-sensitive because fresh leaves contain bioactive compounds, including catechins and polyphenols, that are vulnerable to uncontrolled oxidation and quality degradation when processing is delayed. Financial literacy is often treated as a general managerial capability. In perishable food systems, however, it may also have indirect technical relevance. It can support a firm’s ability to finance timely procurement, labor payments, energy use, maintenance, and technological investment required for continuous processing. Nevertheless, because the present study does not directly measure biochemical or sensory quality indicators, this relationship is treated as a contextual mechanism rather than a directly tested empirical pathway. This argument aligns with SDG-oriented discussions on resilient agricultural production systems and responsible production. However, the study’s empirical focus remains limited to firm-level financial capability, liquidity, and payment-related operational continuity [24,60].

### 2.7. Methodological Limitations and Research Gaps

The existing literature exhibits notable limitations regarding the nexus between finance and food technology. Most studies rely on cross-sectional data, which complicates causal inference on the relationship between financial literacy and operational performance [61,62]. Research has also failed to isolate applied competency from theoretical knowledge [63,64]. Contextually, there is a distinct lack of empirical modeling testing these constructs within the structural confines of the tea sector—an industry where cash flow constraints may influence the risk of quality deterioration in the final food product [6,23]. This study directly addresses these gaps.

## 3. Materials and Methods

### 3.1. Research Design

**Scheme 1:**
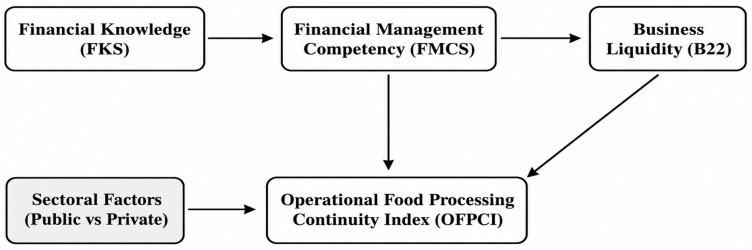
Conceptual model of financial capability, liquidity, and processing continuity.

This study employs a quantitative, cross-sectional design to examine the financial capabilities of tea-processing managers in Rize Province, Türkiye, and their effects on business liquidity and operational food-processing continuity. Scheme 1 summarizes the conceptual model. The model positions financial knowledge (FKS) as a theoretical capability that may influence operational outcomes through financial management competency (FMCS). On the other hand, business liquidity (B22) functions as a key channel through which firms sustain processing efficiency and finance food safety-related investments. In this framework, financial literacy is not treated merely as a stock of theoretical knowledge but as a multidimensional capability that shapes managerial decision-making and operational resilience, consistent with the broader financial literacy perspective advanced in prior studies [24,65]. Structured survey data were collected using the OECD/International Network on Financial Education (INFE) [66] framework and validated psychometric instruments [34,67,68]. The methodology follows the approach employed by ref. [69] for sustainability research in agricultural food enterprises.

### 3.2. Study Area and Sampling

The target population consists of all 86 tea processing plants operating in Rize Province—the most geographically concentrated tea production region in Türkiye, responsible for approximately 68% of the country’s green tea output [8]. Among these plants, 33 (38.4%) are public enterprises affiliated with Çaykur (General Directorate of Tea Enterprises, Rize, Türkiye), and 53 (61.6%) are privately owned. To ensure proportional representation of both public and private sector enterprises, a stratified sampling approach was employed [11]. Between 1 and 4 senior managers were interviewed at each plant, yielding a final analytical sample of 203 respondents: 102 from public enterprises and 101 from private-sector firms. Respondents included business owners, plant managers, accounting managers, and financial managers—individuals directly involved in operational and financial decision-making that impacts processing workflows. This sample structure provides a representative dataset for analyzing the sector, given Rize’s central role in Türkiye’s tea production and food supply chain [3].

### 3.3. Variable Definitions

All key constructs were operationalized through composite indices derived from the survey. The questionnaire was structured into four domains: (i) demographic and firm characteristics; (ii) financial knowledge items (K1–K10); (iii) financial knowledge sources (K11–K16); and (iv) management practices, including liquidity for operational continuity (A1–A8, B1–B28). Table 1 summarizes the variables.

Including OFPCI enables the empirical model to examine payment-related operational continuity as an intermediate firm-level indicator. Because OFPCI is constructed as the reverse-coded counterpart of selected CFDI items, it should not be interpreted as an independently observed physical measure of operational resilience, food safety, or post-harvest loss. Rather, it captures the perceived absence of payment-related disruptions across key processing inputs.

### 3.4. Model Specification

All econometric models are formulated as multiple linear regression specifications. Five models are estimated, each addressing a distinct dependent variable, with model formulations as follows:

Model 1 determines the financial knowledge score (FKS) (Equation (1)):

(1) $$FKS=\beta_{0}+\beta_{1}\cdot Education+\beta_{2}\cdot Experience+\beta_{3}\cdot Public+\beta_{4}\cdot FISI\;+\beta_{5}\cdot FinEducation+\varepsilon$$

The composite FKS is computed from ten knowledge items, where K_1_–K_8_ are binary (correct = 1, incorrect = 0), K_9_ is a 7-point self-assessment item normalized to (0, 1), and K_10_ is a 12-point matching score normalized to (0, 1) [34,79,80]. The resulting FKS ranges from 0 to 10.

Model 2 assesses financial management competency score (FMCS) (Equation (2)):

(2) $$FMCS=\beta_{0}+\beta_{1}\cdot FKS+\beta_{2}\cdot A1+\beta_{3}\cdot A2+\beta_{4}\cdot FAFI+\beta_{5}\cdot FSUS+\varepsilon$$

FMCS is computed as the unweighted mean of five self-assessed management skills (budget, debt, risk, planning, and investment management; B17–B21), each rated on a 7-point Likert scale [24].

Model 3 evaluates the predictors of business liquidity (B22), a proxy for the firm’s perceived operational funding capacity (Equation (3)):

(3) $$Liquidity=\beta_{0}+\beta_{1}\cdot FKS+\beta_{2}\cdot FMCS+\beta_{3}\cdot Public+\beta_{4}\cdot FAFI+\varepsilon$$

Model 4 analyses cash-flow difficulties (CFDI), which may restrict raw material procurement and technological upgrades (Equation (4)):

(4) $$CFDI=\beta_{0}+\beta_{1}\cdot FKS+\beta_{2}\cdot FMCS+\beta_{3}\cdot Liquidity+\beta_{4}\cdot FinAccess+\beta_{5}\cdot Public+\varepsilon$$

CFDI is defined as the binary sum of payment-difficulty indicators across five expenditure categories (raw materials, labor, energy, maintenance, and investment), each ranging from 0 to 5 [25].

Model 5 analyses operational food-processing continuity using OFPCI, a reverse-coded payment-continuity index derived from payment-difficulty items. The index reflects perceived payment-related continuity across key operational inputs rather than independently measured physical processing continuity [76,81]. Therefore, Model 5 should be interpreted as examining associations between financial capability, liquidity, and perceived payment continuity, not as establishing causal effects on objective food-processing performance (Equation (5)):

(5) $$OFPCI=\beta_{0}+\beta_{1}\cdot FMCS+\beta_{2}\cdot B_{22}+\beta_{3}\cdot FKS+\varepsilon$$

### 3.5. Estimation Technique

All regression models were estimated using Ordinary Least Squares (OLS), consistent with the continuous or composite structure of the dependent variables used in the empirical models. Comparative analyses between public and private-sector subsamples were conducted using independent-samples Welch *t*-tests for continuous variables and Chi-square (χ^2^) tests for categorical variables. The mediation hypothesis was tested following the four-step procedure proposed by ref. [82], supplemented by the Sobel test to assess the statistical significance of indirect effects. Overconfidence was quantified by computing the standardized difference between self-assessed financial literacy (A1, rescaled to the FKS range) and objectively measured financial knowledge. Data were analyzed using IBM SPSS Statistics, version 29.0.2.0 (IBM Corp., Armonk, NY, USA) and Python, version 3.10 (Python Software Foundation, Wilmington, DE, USA) statistical libraries.

### 3.6. Diagnostic Tests and Robustness Checks

The structural validity of the financial literacy scale was assessed via Exploratory Factor Analysis (EFA) using Principal Component Analysis with Varimax rotation, consistent with the profiling methodology employed by Ref. [83]. Data suitability was verified using the Kaiser–Meyer–Olkin (KMO) measure of sampling adequacy (threshold: KMO > 0.80) and Bartlett’s Test of Sphericity (*p* < 0.001). Internal consistency reliability was evaluated using Cronbach’s Alpha.

For each regression model, multicollinearity was assessed using the Variance Inflation Factor (VIF), and residual autocorrelation was examined using the Durbin–Watson statistics. To further evaluate the stability and empirical validity of the estimated models, additional robustness and sensitivity checks were conducted. Influential observations were assessed using Cook’s Distance, while 5000-resample bootstrap estimations with bias-corrected and accelerated confidence intervals were employed to examine the robustness of the key coefficients. For models involving Likert-type or ordinal dependent variables, ordinal logistic regression was additionally estimated as a sensitivity check to assess whether the substantive findings were consistent under an alternative model specification.

## 4. Results

### 4.1. Descriptive Statistics and Sector Comparison

**Figure 1:**
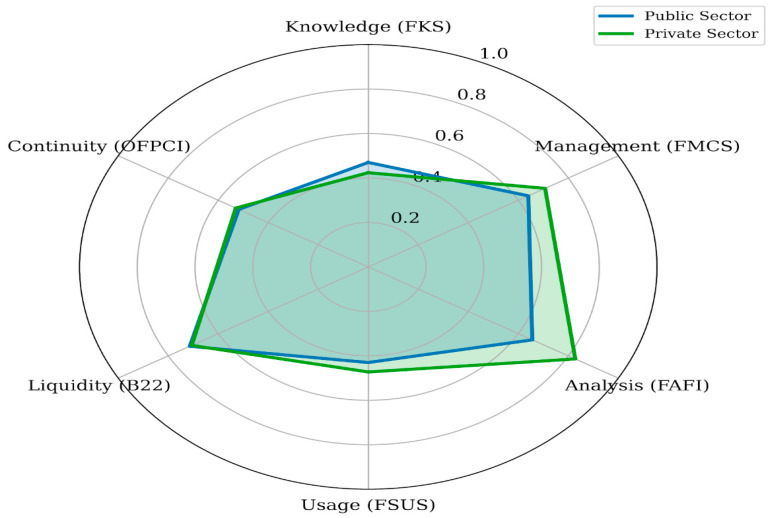
Sectoral comparison of financial literacy and operational resilience indicators.

Figure 1 presents the sectoral comparison of financial literacy and operational resilience indicators and visualizes the “knowledge–practice divergence” between public and private enterprises. Overall, the sample exhibits moderate-to-high levels of financial management competency (FMCS = 4.71/7) and operational liquidity (B22 = 3.55/5). In contrast, the mean FKS remains relatively low (FKS = 4.47/10), indicating persistent deficiencies in theoretical financial literacy compared with the OECD/INFE [66] benchmark of 6.2. Sectoral comparisons show statistically significant differences in FKS (*p* = 0.037), FMCS (*p* = 0.001), and FAFI (*p* = 0.031). However, financial statement usage, information-source diversity, cash-flow difficulties, operational continuity, liquidity, and access to finance do not differ significantly across ownership types (*p* > 0.05).

The public sector records higher theoretical financial knowledge than the private sector (FKS: 4.70 vs. 4.24), which is consistent with its higher participation in formal financial training (63.7% vs. 46.5%; χ^2^ = 5.389, *p* = 0.020). It also displays a marginally stronger liquidity profile (B22: 3.57 vs. 3.53). By contrast, private enterprises report significantly higher financial management competency (FMCS: 4.95 vs. 4.48) and financial analysis frequency (FAFI: 4.14 vs. 3.28), suggesting that competitive market pressures encourage more proactive managerial monitoring and decision-making practices, in line with previous evidence [24]. However, the absence of significant differences in CFDI (2.41 vs. 2.35; *p* = 0.711), OFPCI (2.59 vs. 2.65; *p* = 0.711), B22 (*p* = 0.802), and B23 (*p* = 0.291) indicates that both ownership groups face broadly similar structural constraints in sustaining uninterrupted food-processing continuity. Thus, Figure 1 shows that public enterprises are relatively stronger in knowledge and liquidity-related dimensions, whereas private enterprises are more advanced in managerial competence and analytical practice.

#### 4.1.1. Financial Knowledge Item Analysis

**Figure 2:**
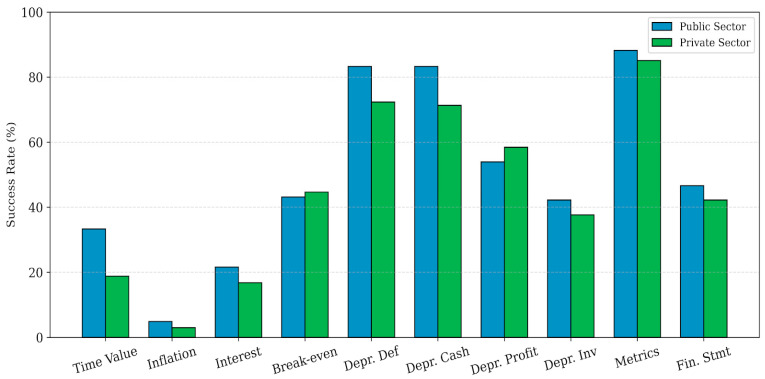
Correct Response Rates for Financial Knowledge Items by Sector.

The results reveal deficiencies in foundational financial concepts, as reflected in the mean FKS of 4.47/10. The weakest areas are the time value of money (26.1%) [84], inflation (3.9%), and interest-rate mechanisms (19.2%) (see Figure 2). In addition, 85% of managers overestimate their financial literacy, suggesting a pervasive overconfidence pattern. Together, these weaknesses may pose potential risks to operational stability in the tea-processing sector. FMCS emerges as the dominant predictor of perceived business liquidity (*β* = 0.336, *p* < 0.001). At the same time, mediation results confirm that financial knowledge and management competency operate as independent constructs, with no indirect effect of FKS on liquidity. Despite this, the near-universal incidence of raw material payment difficulties (93.6%) indicates a structural cash-flow challenge that transcends firm-level capabilities. These constraints may delay tea processing, thereby increasing the risk of leaf degradation and quality deterioration. Since the study does not directly observe processing delays or biochemical quality indicators, this interpretation should be understood as a theoretically grounded implication rather than a directly tested empirical result. The finding highlights the potential relevance of procurement-finance mechanisms for SDG-oriented policies concerned with resilient production and food-loss prevention [85].

The results also reveal an uneven knowledge profile. Managers perform better on routine accounting concepts, particularly the definition of depreciation (K5: 77.8%) and depreciation–cash flow effects (K6: 77.3%), but show weaker performance in forward-looking financial reasoning, such as discounting, inflation adjustment, and interest rate valuation. Public-sector managers generally outperform private-sector managers, possibly reflecting stronger institutional training and administrative routines, consistent with ref. [86]. However, the very low inflation rate in both sectors indicates a sector-wide deficiency.

Overall, Figure 2 suggests that financial knowledge gaps are concentrated in areas directly related to liquidity planning, cost projection, investment appraisal, and operational continuity. As ref. [87] note, such weaknesses may increase SME vulnerability by limiting managers’ ability to anticipate financial stress and respond to changing cost conditions, thereby affecting timely payments for raw materials, machinery upgrades, and continuous processing required for food quality and safety.

#### 4.1.2. Overconfidence in Financial Self-Assessment

A mean overconfidence gap of 1.57 standardized units is observed between self-assessed financial literacy and objectively measured FKS, with approximately 85% of respondents overestimating their financial knowledge relative to their actual performance. Private sector managers exhibit a marginally higher overconfidence bias (Δ = +1.71) compared to public sector counterparts (Δ = +1.42), although this difference is not statistically significant (Welch t = −1.40, *p* = 0.164). This misalignment may increase the risk of suboptimal financial planning and underestimation of liquidity constraints, particularly during peak procurement periods. However, because the present study does not directly observe food safety outcomes, this implication should be interpreted as a potential operational risk rather than a directly tested effect. The findings are consistent with the overconfidence–risk behavior nexus documented in prior studies [45,46] and align with the Dunning-Kruger effect, whereby low-competence individuals disproportionately overestimate their abilities [87].

### 4.2. Correlation Analysis

FMCS is strongly correlated with business liquidity (r = 0.334, *p* < 0.001), highlighting the association between financial management competency and perceived operational funding capacity (Table 2). FMCS is negatively associated with difficulty accessing external finance (B23: r = −0.149, *p* < 0.05), indicating that higher competency is associated with lower perceived difficulty in accessing external finance. This result may also reflect stronger financial preparedness or more effective interaction with financial institutions [72]. In contrast, FKS is not significantly correlated with FMCS, liquidity, CFDI, or OFPCI, suggesting that theoretical financial knowledge alone does not ensure operational continuity and that financial knowledge and applied management competency operate through partially independent pathways.

The inclusion of OFPCI further clarifies the payment-related operational continuity mechanism. OFPCI is positively and significantly correlated with both FMCS (r = 0.194, *p* < 0.05) and business liquidity (B22: r = 0.315, *p* < 0.001), indicating that stronger managerial competency and liquidity capacity are associated with higher payment-related continuity in food-processing operations. This pattern provides preliminary correlational support for Model 5. As OFPCI is the reverse-coded counterpart of payment difficulties across B24–B28, its perfect negative correlation with CFDI (r = −1.00, *p* < 0.001) is conceptually expected. It confirms that the two indices capture opposite sides of the same operational-financial constraint: difficulty versus continuity. This perfect inverse relationship also implies that OFPCI should be interpreted cautiously: it does not represent an independent operational-performance construct, but rather a complementary expression of payment-related continuity versus difficulty.

### 4.3. Regression Results

#### 4.3.1. Model 1: Determinants of Financial Knowledge Score

Work experience is the sole statistically significant predictor of FKS (*β* = 0.018, *p* = 0.041), indicating that each additional year of professional experience increases FKS scores by approximately 0.018 points (Table 3). Public sector affiliation exerts a positive, though marginally non-significant effect on FKS (*β* = 0.403, *p* = 0.105), partially supporting the argument that institutional structures may contribute to higher financial literacy levels [11]. Similarly, the Financial Information Sources Index (FISI) approaches statistical significance (*β* = 0.230, *p* = 0.065), consistent with the multi-channel learning framework proposed by prior studies [74]. In contrast, formal education level and participation in financial training programs do not exert significant direct effects on FKS (both *p* > 0.05). The low overall model fit (R^2^ = 0.074) suggests that financial knowledge is influenced by additional unobserved factors beyond formal education, including domain-specific interest, experiential learning, and the quality of intra-firm training practices [88].

#### 4.3.2. Model 2: Determinants of Financial Management Competency

Confidence in interpreting financial statements (A2) is a strong, statistically significant predictor of applied financial management competency (*β* = 0.277, *p* = 0.004). In contrast, theoretical FKS does not exert a significant effect (*p* = 0.462) (Table 4). These findings indicate that perceptual and self-evaluative dimensions of financial capability can play a more decisive role in operational financial management than objective theoretical knowledge alone. This interpretation is consistent with prior literature emphasizing confidence-based financial behavior and managerial decision-making stages in food-processing and SME contexts [24,45,72].

#### 4.3.3. Model 3: Determinants of Business Liquidity (Processing Continuity)

FMCS emerges as the dominant and highly significant predictor of business liquidity (*β* = 0.336, *p* < 0.001), indicating that a one-unit increase in financial management competency is associated with a 0.336-point increase in perceived liquidity (Table 5). This relationship is particularly critical in the food processing sector, where liquidity is essential for procuring fresh tea leaves and financing processing utilities. In contrast, FKS yields a statistically significant but negative coefficient (*β* = −0.084, *p* = 0.042), suggesting that managers with higher technical financial knowledge tend to evaluate their operational conditions more conservatively and realistically. This pattern reflects a calibration effect, in which financially knowledgeable individuals correct for the overconfidence bias more commonly observed among less knowledgeable counterparts [20,89]. The relatively higher model fit (R^2^ = 0.139) compared to previous models further confirms the central role of applied financial management competency in determining liquidity capacity within the food-sector agricultural context.

#### 4.3.4. Model 4: Determinants of Cash Flow Difficulties

Model 4 fails to attain overall statistical significance (F = 0.571, *p* = 0.722), and no individual predictor achieves significance (all *p* > 0.05) (Table 6).

The model’s negligible explanatory power (R^2^ = 0.014) suggests that the selected firm-level variables do not meaningfully explain variation in cash-flow difficulties. This pattern is consistent with the interpretation that seasonal and sector-wide financing constraints may shape cash-flow problems. However, the non-significant model should not be interpreted as definitive evidence of structural causality. Alternative explanations, including omitted variables, limited variation in CFDI, measurement constraints, and the cross-sectional design of the study, should also be considered. The finding that 93.6% of firms report difficulties in paying for raw materials, particularly fresh tea leaves, during the procurement cycle further suggests that procurement finance is a sector-wide vulnerability. Such liquidity disruptions may delay procurement schedules and increase the risk of fresh leaf deterioration before processing, but the effects on physical quality are not directly measured in this study [25,32].

**Figure 3:**
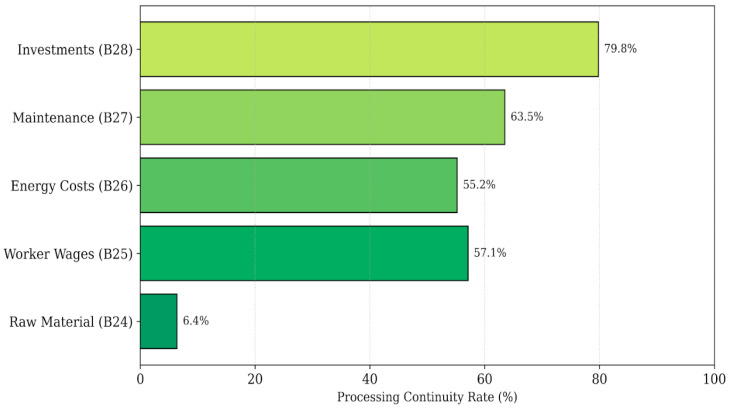
Operational continuity rates across OFPCI components.

Figure 3 further illustrates the component-level structure of OFPCI by reporting the share of firms maintaining payment-related operational continuity across raw material procurement, labor payments, energy costs, maintenance, and investment expenditures. The most critical vulnerability is observed in raw material payments (B24), with only 6.4% of firms reporting continuity, confirming that procurement finance is the weakest point in the tea-processing cycle. This finding is consistent with the view that seasonal liquidity constraints can disrupt production timing and agricultural output [75], and with evidence emphasizing the importance of balanced financial resources for sustaining agricultural production and investment activities [32]. By contrast, higher continuity rates in investment (B28) and maintenance (B27) suggest that firms are relatively better at preserving technical infrastructure than at ensuring uninterrupted raw material procurement. Higher values indicate stronger payment-related operational continuity, not independently observed resilience in physical processing.

#### 4.3.5. Model 5: Determinants of Operational Food Processing Continuity

Model 5 examines the Operational Food Processing Continuity Index (OFPCI), which measures perceived payment-related continuity across raw material procurement, labor payments, energy, technical maintenance, and technological investment. The model is statistically significant overall (F = 15.214, *p* < 0.001) and has moderate explanatory power (R^2^ = 0.204), indicating that payment-related operational continuity is systematically associated with firm-level liquidity and managerial competence (Table 7).

Business liquidity shows the strongest positive association with OFPCI (*β* = 0.298, *p* = 0.001), indicating that firms with stronger perceived cash positions report fewer payment-related disruptions in critical inputs such as fresh tea leaves, labor, energy, and maintenance. This finding is consistent with studies emphasizing the role of liquidity in sustaining agricultural enterprise operations [90]. It also aligns with evidence that seasonal liquidity constraints affect rural labor markets, production timing, and agricultural output [75]. In the tea sector, where fresh leaves must be processed shortly after harvest, such liquidity shortages may increase the risk of procurement delays and operational interruptions.

Financial management competency is also positively and significantly associated with OFPCI (*β* = 0.186, *p* = 0.013), suggesting that budgeting, fund allocation, and cash-flow planning are relevant to the timely financing of operational continuity requirements. This result is consistent with the view that food-sector sustainability depends not only on access to raw materials but also on proactive budgeting for labor, maintenance, and operating expenses [91]. It is also supported by studies that emphasize that agricultural enterprises require a balanced, effectively allocated financial resource structure to sustain production, labor, and investment activities [32].

By contrast, financial knowledge does not have a direct significant association with OFPCI (*β* = −0.058, *p* = 0.169), implying that theoretical knowledge alone is not associated with uninterrupted processing unless translated into managerial action. This interpretation is consistent with the dynamic capabilities’ perspective, which stresses the practical reconfiguration of financial and operational resources under changing conditions [76].

Overall, Model 5 suggests that perceived payment-related processing continuity in the tea sector is more closely associated with liquidity and applied financial management capability than with abstract financial knowledge. From an SDG 12 perspective, these results are relevant to responsible production because liquidity and financial management competency are associated with fewer payment-related operational interruptions. However, the study does not directly measure food loss or resource-use efficiency; therefore, the SDG-related interpretation should be treated as a policy-relevant implication rather than a directly tested outcome. From an SDG 2 perspective, the same findings suggest that liquidity and managerial competency may support resilient agricultural value chains by facilitating the intake of fresh leaves, energy use, labor continuity, and the processing of highly perishable inputs. Thus, financial management competency can be interpreted as an operational capability associated with firm-level financial resilience and broader food-system sustainability goals [13,15,16].

### 4.4. Exploratory Factor Analysis and Reliability

Exploratory Factor Analysis confirmed the suitability of the data structure for dimensional analysis (KMO = 0.837; Bartlett’s χ^2^ = 770.754, df = 66, *p* < 0.001). Principal Component Analysis with Varimax rotation yielded a four-factor solution with eigenvalues greater than one, collectively explaining 64.2% of total variance. The identified dimensions—Financial Management (20.8%), Financial Knowledge (17.1%), Financial Attitude (16.7%), and Financial Behavior (9.6%) are broadly consistent with the multidimensional financial literacy framework proposed in prior literature [65]. The Financial Management factor demonstrates near-perfect internal consistency (Cronbach’s α = 0.909), while the overall scale exhibits acceptable reliability (α = 0.721). The Financial Management factor explains the largest proportion of variance. This result supports the argument that applied financial competency is a central component of financial capability in this agricultural food-sector context. It also suggests that practical financial management skills are relevant to sustaining complex food-processing operations and payment-related operational continuity.

### 4.5. Mediation Analysis

**Figure 4:**
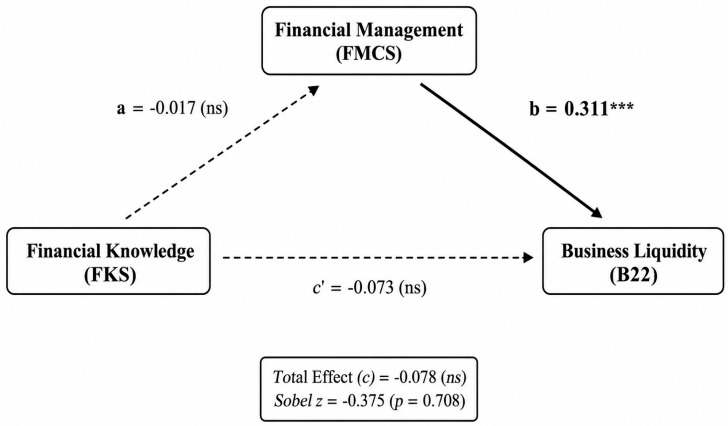
Path analysis of financial knowledge, management competency, and liquidity. Note: *** indicates statistical significance at the 1% level.

Following Baron and Kenny’s [82] four-step procedure, FMCS was tested as a potential mediator in the relationship between FKS and business liquidity. As shown in Figure 4, the total effect of FKS on liquidity is not statistically significant (c = −0.078, ns), and FKS also fails to predict FMCS significantly (a = −0.017, ns). Although FMCS has a strong and significant positive effect on liquidity (b = 0.311, *p* < 0.001), the Sobel test indicates no statistically significant indirect effect (z = −0.375, *p* = 0.708). These findings indicate that FMCS does not mediate the FKS–liquidity relationship. Instead, financial management competency appears to operate as an independent predictor of liquidity, reinforcing prior evidence that practical operational capabilities, rather than theoretical financial knowledge alone, are more critical for maintaining liquidity and preventing disruptions in food processing and supply chain continuity [24,40,43,72].

### 4.6. Robustness and Sensitivity Checks

To assess the empirical stability of the five baseline models, we conducted several robustness and diagnostic checks. First, multicollinearity diagnostics indicated that the estimated models were not affected by problematic collinearity among independent variables. The VIF values ranged between 1.04 and 2.12 across the models, remaining well below the commonly accepted threshold of 5.0 and the more conservative threshold of 3.0. Second, the Durbin–Watson statistics ranged from 1.56 to 1.84, suggesting no serious residual autocorrelation. Third, Cook’s Distance values did not exceed 0.08 in any model, remaining far below the conventional critical threshold of 1.0. This indicates that highly influential individual observations did not drive the regression estimates.

The robustness of the main OLS findings was further evaluated using 5000-resample bootstrap estimations with bias-corrected and accelerated confidence intervals. The bootstrap results confirm the stability of the key predictors across the main models. In Model 1, work experience remains a significant determinant of FKS. In Model 2, confidence in interpreting financial statements remains a robust predictor of FMCS. In Model 3, financial management competency remains a highly significant determinant of business liquidity. Model 4 remains statistically insignificant under the bootstrap procedure, supporting the interpretation that cash flow difficulties reflect structural and sector-wide constraints rather than firm-level managerial deficiencies. In Model 5, business liquidity remains the strongest and statistically significant determinant of OFPCI, confirming the robustness of the link between liquidity capacity and operational food-processing continuity.

As an additional sensitivity check, ordinal logistic regression was estimated for models involving Likert-type or ordinal dependent variables. The results are consistent with the OLS findings. In Model 3, the positive effect of FMCS on business liquidity remains statistically significant under the ordinal specification, indicating that the central conclusion does not depend on the linear OLS framework. Similarly, for Model 5, business liquidity continues to have the strongest effect on operational food-processing continuity, supporting the interpretation that liquidity is a key condition for maintaining uninterrupted processing operations. Overall, these robustness and sensitivity checks strengthen confidence in the empirical validity of the main findings. They also support the argument that applied financial management competency and liquidity resilience are central to payment-related food-processing continuity. The full diagnostic and robustness statistics are presented in Appendix A (Table A1).

## 5. Discussion

### 5.1. Financial Competency as an Operational Capability Associated with Liquidity

The findings indicate that financial management competency is significantly associated with perceived business liquidity, suggesting that applied financial capability may play an operational role in supporting processing continuity in tea-processing enterprises. The strong FMCS–liquidity association observed in Model 3 (*β* = 0.336, *p* < 0.001) is consistent with the KBV/RBV perspective that applied financial management competency can function as an intangible strategic asset [20,38]. The four-factor EFA structure also supports the multidimensional financial literacy framework proposed by refs. [65,67]. In this structure, the Financial Management dimension explains the largest share of variance (20.8%), exceeding the Financial Knowledge dimension (17.1%). This finding suggests that applied competency is the most salient component of financial capability in this agricultural food-sector context. Consistent with prior literature [24], the findings further suggest that the ability to translate theoretical knowledge into practical financial management decisions is relevant to business sustainability, particularly in securing operational funding, evaluating technological upgrades, and sustaining payment-related continuity necessary for processing activities.

### 5.2. The Financial Knowledge Deficit and Food Quality Implications

The FKS of 4.47/10 falls below the OECD/INFE benchmark of 6.2 [60] and comparable agricultural contexts, including Sri Lankan tea production, Kenyan smallholder coffee farming, and Indonesian agricultural enterprises [1,92,93]. The most critical deficit concerns the time value of money (26.1% correct), regarded as the “ABC of financial literacy” [84]. Moreover, limited understanding of inflation (3.9%) and interest rates (19.2%) may weaken pricing, borrowing, and liquidity management decisions in a sector highly exposed to input-cost volatility. These weaknesses may indirectly increase operational vulnerability during procurement and processing periods. In highly perishable tea-processing chains, processing delays may increase the risk of quality deterioration [3,15]. However, because this study does not directly measure biochemical quality or physical post-harvest losses [84], this interpretation should be treated as a contextual implication rather than a directly observed empirical outcome.

The findings further indicate that formal financial training attendance exerts a negligible effect on FKS, consistent with Collins and Holden’s [94] argument that short-duration programs often generate limited measurable impact. Conversely, the positive marginal effect of the Financial Information Sources Index (FISI) supports Filbeck et al.’s [74] multi-channel learning hypothesis, suggesting that broader and continuous access to financial information incrementally enhances financial knowledge. Effective financial interventions should therefore be viewed not only as literacy-enhancing mechanisms but also as essential components of food quality control and supply chain resilience, requiring applied, sector-specific, and sustained learning environments rather than one-off theoretical training programs [95,96].

### 5.3. Overconfidence and the Calibration Problem in Food Processing

The overconfidence gap observed among 85% of managers, with a mean miscalibration of 1.57 standardized units, has potential implications for financial planning and operational risk management in the tea-processing sector [45,46]. Prior research links overconfidence to suboptimal financial decision-making through the Dunning–Kruger effect, whereby individuals with lower competence tend to overestimate their capabilities. In the context of agricultural food processing, such miscalibration may lead managers to underestimate liquidity vulnerabilities and overestimate their operational preparedness, increasing the risk of sudden cash shortages during peak processing periods. Such disruptions may increase the likelihood of processing delays during peak seasons, although the present data do not allow direct measurement of spoilage or product quality outcomes.

The negative coefficient on FKS in Model 3 (*β* = −0.084, *p* = 0.042) provides evidence of a calibration mechanism, indicating that managers with higher objective financial knowledge evaluate liquidity conditions more conservatively and realistically. This finding suggests that realistic self-assessment is critical for planning continuous, safe processing workflows, particularly in sectors that depend on uninterrupted raw material processing and strict quality preservation standards. More broadly, the results also highlight the limitations of relying solely on self-reported liquidity assessments in environments characterized by widespread managerial overconfidence [87].

### 5.4. Knowledge–Competency Decoupling and Policy Implications

The findings suggest that financial knowledge and financial management skills represent partially independent constructs with distinct determinants, rather than sequentially linked dimensions of financial literacy [24,45]. Specifically, knowledge appears to be accumulated primarily through professional experience and diversified information sourcing, whereas applied management competency is more strongly associated with confidence and self-efficacy perceptions. The absence of a significant direct relationship between FKS and FMCS therefore challenges the assumption, common in much of the financial literacy literature, that theoretical knowledge automatically translates into managerial capability. This decoupling carries important implications for agricultural extension programs and food system capacity-building initiatives [43].

Policy interventions aimed at strengthening food-sector resilience should not focus exclusively on increasing theoretical financial literacy; they must also separately and explicitly develop practical cash flow management and operational decision-making skills. Such applied competencies are closely associated with firms’ ability to sustain liquidity, maintain processing operations, and preserve food-processing standards within time-sensitive agricultural supply chains.

### 5.5. Cash-Flow Difficulties: Sector-Wide Constraints and Alternative Explanations

The non-significant Model 4 results indicate that the selected firm-level variables do not explain meaningful variation in CFDI (R^2^ = 0.014, *p* = 0.722). This pattern is compatible with the interpretation that cash-flow difficulties may be influenced by seasonal procurement cycles and sector-wide financing conditions. Nevertheless, the findings should be interpreted cautiously, as non-significant regression results cannot, in themselves, establish structural causality. Omitted variables, measurement limitations, limited within-sample variation, and the cross-sectional design may also contribute to the model’s weak explanatory power. With 93.6% of enterprises reporting difficulties paying for raw materials, particularly fresh tea leaves, procurement finance appears to be a widespread vulnerability in the sector. However, the causal source of this vulnerability requires further investigation using longitudinal, objective financial data [25,32].

The findings imply that improving individual-level financial literacy alone may be insufficient to address payment-related disruptions in tea processing operations without broader procurement financing mechanisms, state support programs, and coordinated payment scheduling systems [3,31]. When processors cannot secure timely financing, procurement delays may interrupt continuous processing workflows and increase the risk of fresh leaf deterioration before production. Consequently, maintaining processing continuity and food quality in this sector requires systemic financial and institutional solutions that sustain production-line operations, rather than relying solely on individual capacity-building interventions.

### 5.6. Indirect Implications for Food-System Resilience and SDG-Oriented Policy

The empirical evidence from this study suggests that financial management competency is associated with liquidity and payment-related processing continuity, which may have indirect relevance for food-system resilience in highly perishable agro-food chains. The significant association between FMCS and liquidity, and the positive association between liquidity and OFPCI, indicate that financial resilience is not merely an internal business capability but a potential mechanism for sustaining payment-related processing flows. This interpretation aligns with ref. [13]’s conceptualization of food system resilience as the capacity to maintain sufficient, appropriate, and accessible food provision despite disturbances. It is also consistent with Ref. [14]’s argument that local food system resilience is critical for protecting food security under economic and institutional shocks.

From the perspective of SDG 12, the findings are relevant to discussions on food loss prevention, as payment-related disruptions may increase the risk of processing delays. Ref. [17] emphasizes that food losses occur across the post-harvest, processing, and distribution stages, whereas the present study shows that liquidity shortages may delay fresh leaf procurement, interrupt energy and labor payments, and weaken maintenance capacity in tea-processing facilities. However, the study does not directly measure physical food loss, resource-use efficiency, or post-harvest waste; therefore, the SDG 12 interpretation should be framed as an indirect policy implication. From the perspective of SDG 2, operational continuity in tea-processing facilities can help stabilize agricultural value chains, particularly in regions where tea production supports rural livelihoods. Nevertheless, the present study does not directly measure food availability, nutrition, or household food-security outcomes. Consistent with the Ref. [18] call for a global food-security narrative toward 2030, this study provides a micro-level empirical pathway linking financial management competency, liquidity resilience, payment-related processing continuity, and sustainable food-system policy discussions.

### 5.7. The Public–Private Divergence: Complementary Advantage

Public sector enterprises hold a systematic advantage in theoretical financial knowledge (FKS: 4.70 vs. 4.24, *p* = 0.037) and participation in formal training (63.7% vs. 46.5%, *p* = 0.020), largely attributable to Çaykur’s institutionalized human capital development infrastructure. In contrast, private sector firms exhibit significantly higher applied financial management competency (FMCS: 4.95 vs. 4.48, *p* = 0.001), more frequent financial analysis practices, stronger risk management behavior (*p* = 0.004), and greater proactivity in seeking alternative financing sources (16.8% vs. 3.9%, *p* < 0.001). These patterns reflect the competitive pressures private processors face to sustain operational efficiency and continuously invest in effective processing technologies along the food production chain [32].

At the same time, the absence of significant sectoral differences in liquidity conditions, access to financing, and cash flow difficulties suggests that financial performance outcomes are shaped more strongly by macro-structural and sector-wide constraints than by ownership-specific managerial capabilities alone. The findings therefore support an integrative policy approach that combines the institutionalized knowledge infrastructure and training capacity of public enterprises with the operational dynamism, managerial adaptability, and proactive financial practices of private firms. Such integration appears essential for strengthening overall food system resilience, sustaining processing continuity, and preserving food quality standards across the tea sector.

## 6. Conclusions

### 6.1. Principal Contributions

This study makes substantive contributions to financial literacy, agricultural enterprise, as well as the literature on sustainable food systems. First, it provides empirical evidence from the Turkish tea sector showing that applied financial management competency is significantly associated with perceived business liquidity. This liquidity capacity represents an important intermediate condition for maintaining payment-related processing continuity in highly perishable tea-processing chains. Second, it extends the analysis beyond traditional agricultural economics by showing that structural cash-flow constraints, particularly delayed raw material payments, may increase the risk of processing interruptions and quality deterioration, although physical quality outcomes are not directly measured in this study. Third, it links firm-level financial capability to SDG 2 and SDG 12 discussions by suggesting that liquidity resilience and payment-related operational continuity may be relevant intermediate mechanisms for sustainable agro-food processing. Finally, by integrating financial management competency into debates on food-system resilience, the study contributes to the broader food-security literature that emphasizes the need for resilient, sustainable, and inclusive food systems by 2030 [13,14,18].

### 6.2. Summary of Key Findings

Deficiencies in foundational financial concepts (mean FKS: 4.47/10), particularly in the time value of money (26.1%), inflation (3.9%), and interest rate mechanisms (19.2%), together with pervasive overconfidence (85% of managers overestimating their financial literacy), pose significant risks to operational stability in the tea processing sector. FMCS emerges as the dominant determinant of business liquidity (*β* = 0.336, *p* < 0.001), while mediation results confirm that financial knowledge and management competency operate as independent constructs with no indirect effect from FKS to liquidity. Despite this, the near-universal incidence of raw material payment difficulties (93.6%) indicates a structural cash flow crisis that transcends firm-level capabilities. These constraints may delay tea processing and thereby increase the risk of leaf degradation and quality deterioration. Since the study does not directly observe processing delays or biochemical quality indicators, this interpretation should be understood as a theoretically grounded implication rather than a directly tested empirical result. This finding highlights the potential relevance of procurement-finance mechanisms for SDG-oriented policies concerned with resilient production and food-loss prevention.

### 6.3. Policy Recommendations

To strengthen the economic sustainability of the tea sector and support processing continuity under seasonal liquidity constraints, a dual-track policy is required. In the short term, applied training should prioritize cash-flow planning and broader practical management skills, such as budgeting and financial statement interpretation [95,97]. These programs may reduce the risk of sudden payment-related disruptions. This priority is particularly important because managerial competency, rather than theoretical knowledge, is associated with liquidity outcomes. The high unmet demand for financial training (80.9% of non-participants expressing willingness to participate) further indicates substantial latent capacity for rapid uptake of such applied skills.

In the medium term, reducing the risk of processing delays and potential post-harvest deterioration requires structural interventions that can be conceptualized as procurement-continuity finance. These include subsidized working-capital credit for fresh leaf procurement, state-supported payment guarantee schemes, extended seasonal financing mechanisms, and digital FinTech systems enabling real-time cash-flow monitoring and real-time payments to farmers, thereby facilitating timely leaf processing [51,52,58,90]. Such instruments may support SDG 12.3-oriented policy objectives by reducing payment-related disruptions that can contribute to processing delays and potential quality deterioration. They may also support SDG 2.4-oriented objectives by sustaining more resilient agricultural production systems. In addition, collaborative platforms that align Çaykur’s institutional knowledge infrastructure with the operational dynamism of private enterprises may be associated with enhanced sectoral financial resilience. Blockchain-based supply-chain financing may also be associated with reduced payment delays and greater transparency across the tea value chain [3]. Investments in quality-preserving infrastructure, including controlled-withering units, energy-continuity systems, maintenance financing, and, where technically appropriate, cold-chain or controlled-storage solutions, should also be evaluated within feasibility, governance, and institutional-capacity constraints.

### 6.4. Limitations

The cross-sectional design precludes causal identification of the examined relationships, and reliance on self-reported data introduces susceptibility to bias, including social desirability effects and systematic miscalibration associated with observed overconfidence. External validity is confined to the Rize Province tea processing sector, limiting generalizability to comparable agricultural or resource-processing industries with similar structural characteristics, particularly those involving highly perishable inputs. In addition, the study does not directly measure physical post-harvest losses, biochemical quality indicators, food safety outcomes, or household-level food security indicators. Therefore, references to food quality, food-loss prevention, SDG 2, and SDG 12 should be interpreted as indirect policy-relevant implications rather than directly tested empirical outcomes. Moreover, OFPCI is constructed as a reverse-coded payment-continuity index based on selected cash-flow difficulty items; thus, it captures perceived payment-related operational continuity rather than independently observed processing performance. Future research should combine survey-based financial capability measures with objective processing, quality, and food-loss data to validate these mechanisms.

### 6.5. Directions for Future Research

Future research should link firm-level financial data directly with physical food quality metrics, such as biochemical analysis of processed tea batches, to quantify the extent to which financial delays translate into measurable food degradation [72,74]. This should be complemented by objective firm-level financial records, including balance sheet indicators, audited income statements, and banking data, to validate and strengthen the observed relationships. Longitudinal and panel designs are further required to evaluate the developmental trajectory from financial knowledge acquisition to applied management competency, as well as to assess the sustained effects of structured financial literacy and FinTech-based interventions on food loss reduction [96,98,99]. Collectively, such approaches would enable more robust causal identification and provide stronger empirical grounding for evidence-based food security governance and policy design.

## Figures and Tables

**Table 1:** Variable Definitions and Measurement Scales.

Variable Name	Symbol	Description	Range	References
Financial Knowledge Score	FKS	Normalized sum of K1–K10	0–10	[34]
Financial Management Competency Score	FMCS	Mean of B17–B21 (Likert scale)	1–7	[24]
Financial Analysis Frequency Index	FAFI	Weighted frequency score	0–5	[70,71]
Financial Statement Usage Score	FSUS	Number of financial statements used (B7–B10)	0–4	[72]
Financial Information Sources Index	FISI	Number of information sources used (K11–K16)	0–6	[73,74]
Cash Flow Difficulty Index	CFDI	Sum of payment difficulties impacting processing (B24–B28)	0–5	[25,75]
Operational Food Processing Continuity Index	OFPCI	Reverse-coded payment-continuity index based on B24–B28; higher values indicate fewer payment-related disruptions in raw materials, labor, energy, maintenance, and investment.	0–5	[76]
Business Liquidity	B22	Self-assessment of operational funding capacity (Likert scale)	1–5	[77]
Difficulty Accessing Finance	B23	Self-assessment of external funding access (Likert scale)	1–5	[72,78]

Note: All composite indices were constructed through standardization and weighted aggregation.

**Table 2:** Pearson Correlation Matrix (*n* = 203).

Variable	FKS	FISI	FSUS	FAFI	FMCS	B22	B23	CFDI	OFPCI
FKS	1	—	—	—	—	—	—	—	
FISI	0.127	1	—	—	—	—	—	—	
FSUS	0.215 **	0.249 ***	1	—	—	—	—	—	
FAFI	0.102	0.130	0.261 ***	1	—	—	—	—	
FMCS	−0.026	0.026	−0.020	0.119	1	—	—	—	
B22 (Liquidity)	−0.129	−0.053	−0.019	0.015	0.334 ***	1	—	—	
B23 (Finance)	−0.056	−0.064	−0.102	−0.017	−0.149 *	0.011	1	—	
CFDI	0.099	0.000	−0.014	−0.008	0.013	−0.064	−0.019	1	
OFPCI	−0.099	0.000	0.014	0.008	0.194 *	0.315 ***	0.019	−1.00 ***	1

Note: * *p* < 0.05; ** *p* < 0.01; *** *p* < 0.001.

**Table 3:** OLS Regression Results—Model 1: Determinants of FKS.

Independent Variables	*β*	Std. Error	t	*p*	95% CI
Constant	3.255	0.374	8.704	<0.001 ***	[2.517; 3.992]
Education Level (1–4)	0.112	0.113	0.991	0.323	[−0.111; 0.335]
Work Experience (years)	0.018	0.009	2.056	0.041 *	[0.001; 0.035]
Public Sector (1 = Yes)	0.403	0.247	1.627	0.105	[−0.085; 0.891]
FISI (0–6)	0.230	0.124	1.854	0.065	[−0.015; 0.474]
Financial Education (1 = Yes)	−0.098	0.226	−0.433	0.665	[−0.545; 0.349]

Note: *n* = 203. Dependent variable: FKS (0–10). R^2^ = 0.074; Adjusted R^2^ = 0.050; F (5, 197) = 3.131, *p* = 0.010; Durbin–Watson = 1.80. * *p* < 0.05; *** *p* < 0.001.

**Table 4:** OLS Regression Results—Model 2: Determinants of FMCS.

Independent Variables	*β*	Std. Error	t	*p*	95% CI
Constant	2.987	0.520	5.743	<0.001 ***	[1.961; 4.013]
FKS (0–10)	−0.033	0.045	−0.737	0.462	[−0.123; 0.056]
A1: Literacy Self-Assessment (1–7)	0.150	0.063	2.365	0.019 *	[0.025; 0.275]
A2: Confidence in Reading Financial Statements (1–5)	0.277	0.094	2.935	0.004 **	[0.091; 0.463]
FAFI (0–5)	0.038	0.023	1.653	0.100	[−0.007; 0.083]
FSUS (0–4)	−0.092	0.093	−0.991	0.323	[−0.275; 0.091]

Note: *n* = 203. Dependent variable: FMCS (1–7). R^2^ = 0.086; Adjusted R^2^ = 0.063; F (5, 197) = 3.723, *p* = 0.003; Durbin–Watson = 1.785. * *p* < 0.05; ** *p* < 0.01; *** *p* < 0.001.

**Table 5:** OLS Regression Results—Model 3: Determinants of Business Liquidity (B22).

Independent Variables	*β*	Std. Error	t	*p*	95% CI
Constant	2.219	0.371	5.980	<0.001 ***	[1.487; 2.950]
FKS (0–10)	−0.084	0.041	−2.050	0.042 *	[−0.165; −0.003]
FMCS (1–7)	0.336	0.064	5.258	<0.001 ***	[0.210; 0.463]
Public Sector (1 = Yes)	0.233	0.133	1.757	0.081	[−0.029; 0.494]
FAFI (0–5)	0.002	0.020	0.076	0.940	[−0.039; 0.042]

Note: *n* = 203. Dependent variable: B22 (1–5). R^2^ = 0.139; Adjusted R^2^ = 0.122; F (4, 198) = 8.018, *p* < 0.001; Durbin–Watson = 1.648. * *p* < 0.05; *** *p* < 0.001.

**Table 6:** OLS Regression Results—Model 4: Determinants of Cash Flow Difficulty Index (CFDI).

Independent Variables	*β*	Std. Error	t	*p*	95% CI
Constant	2.140	0.666	3.211	0.002 **	[0.826; 3.454]
FKS (0–10)	0.069	0.057	1.203	0.230	[−0.044; 0.182]
FMCS (1–7)	0.052	0.096	0.545	0.587	[−0.137; 0.242]
B22: Business Liquidity (1–5)	−0.088	0.099	−0.881	0.380	[−0.284; 0.109]
B23: Difficulty Accessing Finance (1–5)	−0.011	0.087	−0.121	0.903	[−0.183; 0.161]
Public Sector (1 = Yes)	0.063	0.185	0.339	0.735	[−0.302; 0.427]

Note: *n* = 203. Dependent variable: CFDI (0–5). R^2^ = 0.014; Adjusted R^2^ = −0.011; F (5, 197) = 0.571, *p* = 0.722; Durbin–Watson = 1.566. ** *p* < 0.01.

**Table 7:** OLS Regression Results—Model 5: Determinants of Operational Food Processing Continuity Index (OFPCI).

Independent Variables	*β*	Std. Error	t	*p*	95% CI
Constant	1.421	0.458	3.102	0.002 **	[0.518; 2.324]
FMCS	0.186	0.074	2.514	0.013 *	[0.040; 0.332]
B22: Business Liquidity	0.298	0.082	3.634	0.001 ***	[0.136; 0.460]
FKS	−0.058	0.042	−1.381	0.169	[−0.141; 0.025]

Note: *n* = 203. Dependent variable: OFPCI (0–5). R^2^ = 0.204; Adjusted R^2^ = 0.192; F (3, 199) = 15.214, *p* < 0.001; Durbin–Watson = 1.780. * *p* < 0.05; ** *p* < 0.01; *** *p* < 0.001.

## Data Availability

The raw data supporting the conclusions of this study are available on request from the corresponding author due to privacy and ethical restrictions related to the survey-based nature of the research.

## Appendix A

A multi-stage robustness assessment was conducted to evaluate the statistical stability of the reported findings. Multicollinearity was assessed using VIF values, residual autocorrelation was evaluated through the Durbin–Watson statistic, and influential observations were examined using Cook’s Distance. In addition, 5000-resample bias-corrected bootstrap estimations were performed to assess the stability of the key coefficients beyond normality-based assumptions. For models involving ordinal or Likert-type dependent variables, ordinal regression was estimated as a sensitivity check. Overall, the diagnostic and robustness checks support the empirical stability of the main findings. The non-significant result for Model 4 is consistent with the interpretation that cash-flow difficulties may reflect sector-wide structural constraints; however, this result should also be considered alongside potential omitted variables, measurement limitations, and the study’s cross-sectional design.

**Table A1:** Robustness and Diagnostic Checks for the Baseline Regression Models.

Model	Dependent Variable	Key Predictor	OLS *p*-Value/Model *p*-Value	Bootstrap *p*-Value	Robustness Status
Model 1	Financial Knowledge Score (FKS)	Work Experience	0.041	0.039	Robust
Model 2	Financial Management Competency Score (FMCS)	Confidence in Reading Financial Statements (A2)	0.004	0.002	Robust
Model 3	Business Liquidity (B22)	Financial Management Competency Score (FMCS)	<0.001	<0.001	Highly robust
Model 4	Cash Flow Difficulty Index (CFDI)	No significant firm-level predictor	F-test *p* = 0.722	F-test *p* = 0.698	Consistent with structural constraint interpretation, not causal evidence
Model 5	Operational Food Processing Continuity Index (OFPCI)	Business Liquidity (B22)	0.001	0.001	Highly robust

Note: Bootstrap results are based on 5000 resamples with bias-corrected and accelerated confidence intervals. VIF values ranged between 1.04 and 2.12; Durbin–Watson statistics ranged between 1.56 and 1.84; and Cook’s Distance values remained below 0.08 across all models.
